# Grafting of Porous Conductive Fiber Mats with an Antifouling Polymer Brush by Means of Filtration‐Based Surface Initiated ATRP

**DOI:** 10.1002/marc.202300069

**Published:** 2023-04-06

**Authors:** Sheung‐Yin Li, Benjamin S. Schon, Jadranka Travas‐Sejdic

**Affiliations:** ^1^ School of Chemical Sciences The University of Auckland 23 Symonds Street, Auckland CBD Auckland 1010 New Zealand; ^2^ The MacDiarmid Institute of Advanced Materials and Nanotechnology Wellington 6140 New Zealand; ^3^ The New Zealand Institute for Plant and Food Research Limited Canterbury Agriculture & Science Centre 74 Gerald St Lincoln 7608 New Zealand

**Keywords:** conductive membranes, filtration‐based graftings, fouling, poly(3,4‐ethylenedioxythiophene), surface‐initiated atom transfer radical polymerizations

## Abstract

This work addresses the challenge of surface modification of porous, electrospun fiber mats containing an insoluble conducting polymer coating. Herein, a novel methodology of grafting a polymer brush onto conducting polymer fiber mats is developed that employs filtering of the polymerization solution through the fiber mat. An electrospun sulfonated polystyrene‐poly(ethylene‐*ran*‐butylene)‐polystyrene (sSEBS) fiber mat is first coated with a layer of conducting copolymer bearing an Atom Transfer Radical Polymerization (ATRP) initiating functionality (PEDOT‐Br). The surface‐initiated ATRP from the fibers’ surface is then carried out to graft a hydrophilic polymer brush (poly(ethylene glycol) methyl ether methacrylate) by means of filtering the polymerization solution through the fiber mat. Scanning electron microscopy (SEM) images reveal a progressive change in the morphology of the fiber mat surface with the increasing volume of the filtrated polymerization solution, while energy dispersive X‐ray spectrosdcopy (EDX) spectra show a change in the atomic oxygen to sulfur (O/S) ratio, therefore confirming the successful grafting from the fibers’ surface. The conductive fiber mat grafted with hydrophilic brushes shows a 20% reduction in the non‐specific adsorption of bovine serum albumin (BSA) compared to a pristine fiber mat. This study is a proof‐of‐concept for this novel, filtration‐based, surface‐initiated polymerization methodology.

## Graphical abstract

A novel filtration methodology is developed to graft a polymer brush onto conducting polymer fiber mats through surface‐initiated atom transfer radical polymerization. A progressive change in the morphology and elemental composition of the fiber mat surface is observed with the increasing volume of the filtrated polymerization solution, with a 20% reduction in the non‐specific adsorption of BSA.

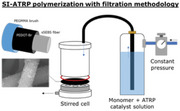

## 1 Introduction

Fouling on filtration membranes can cause long‐term loss of filtration performance and a decrease in the throughput of the filtration system.^[^ ^1^, ^2^, ^3^, ^4^, ^5^ ^]^ Fouling mitigation, therefore, has been one of the ongoing research topics related to filtration membranes. In our previous studies, we have shown that conducting polymer‐coated filtration membranes can provide a convenient means of monitoring and measuring membrane fouling,^[^ ^6^ ^]^ and controlling the flux flow through a membrane.^[^ ^7^ ^]^ Both of these works demonstrated remarkable applications of the conducting polymer coatings in the fouling mitigation in filtration systems through an electrical input and output. However, it would be desirable to further improve the antifouling performance of these conductive membranes.

The grafting of hydrophilic polymer brush on the membrane surface has been shown to be an effective fouling mitigation approach, including through surface‐initiated atom transfer radical polymerization (SI‐ATRP).^[^ ^3^, ^8^, ^9^, ^10^, ^11^ ^]^ The SI‐ATRP approach provides grafting of a broad range of hydrophilic monomers, including sulfobetaine methacrylate,^[^ ^11^ ^]^ 2‐(dimethylamino)ethyl methacrylate,^[^ ^9^, ^12^ ^]^ and poly(ethylene glycol) methyl ether methacrylate (PEGMMA).^[^ ^8^, ^9^, ^10^, ^13^ ^]^ These monomers provide a good antifouling performance against proteins^[^ ^8^, ^10^, ^11^, ^13^ ^]^ and microorganisms^[^ ^9^ ^]^ to maintain the filtration performance of the membranes.^[^ ^8^, ^10^, ^11^ ^]^ Apart from a wide range of monomers available, the hydrophilic polymer brushes can have a high grafting density and varying polymer brush lengths to provide an additional steric hindrance to the incoming foulant to the surface.^[^ ^3^, ^8^, ^9^, ^10^, ^11^ ^]^

The SI‐ATRP on a filtration membrane is facilitated by the introduction of an ATRP initiator on the membrane's surface. In some of the early studies, the fluorine atom of the poly(vinylidene fluoride) membrane directly participated in the activation and de‐activation of the ATRP catalyst.^[^ ^12^, ^13^ ^]^ Later, peroxide and hydroperoxide groups were introduced on the membrane surface through ultraviolet (UV) irradiation and ozone treatment.^[^ ^8^, ^9^, ^10^, ^11^ ^]^ These reactive groups could either directly participate in the polymerization process^[^ ^9^ ^]^ or provide reaction sites for the grafting of ATRP initiators on the membrane surface.^[^ ^8^, ^10^, ^11^ ^]^ The UV/ozone pre‐treatment allowed for carrying out SI‐ATPR on a broader range of membranes, including polypropylene.^[^ ^9^ ^]^

While SI‐ATRP could be used for versatile antifouling modifications of a wide range of membrane substrates with different hydrophilic polymers, the surface morphology of these membranes is usually flat.^[^ ^8^, ^9^, ^10^, ^11^, ^12^, ^13^ ^]^ The ATRP kinetic on these membranes generally fitted very well with the classical ATRP brush‐grafting model on a flat substrate. Brush grafting, by means of SI‐ATRP, on electrospun membranes is very challenging because of the high roughness and the random orientation of the electrospun fibers that comprise the membrane. While some literature reports successful preservation of the morphology of the original fiber mat,^[^ ^14^, ^15^, ^16^ ^]^ others report a loss of the original morphological features^[^ ^16^, ^17^, ^18^ ^]^ and fiber deformation.^[^ ^16^, ^18^ ^]^ In addition, fiber mats containing conducting polymer coatings are difficult to functionalize as the ATRP initiating functionality needs to be introduced through chemical modification of conducting polymer.^[^ ^19^ ^]^ It is also important that SI‐ATRP grafting of polymer brush is achieved throughout the thickness of the fiber mats.

Herein we developed a novel methodology for SI‐ATRP grafting from a conductive polymer‐coated fiber mat surface, exemplified by the grafting of an antifouling polymer brush, where the polymerization solution is filtered repetitively, at constant pressure, through the fiber mat membrane. The fiber mat was prepared by electrospinning of sulfonated polystyrene‐poly(ethylene‐*ran*‐butylene)‐polystyrene (sSEBS) and coated with a layer of conductive poly(3,4‐ethylenedioxythiopehene) (PEDOT) copolymer functionalized with ATRP initiating sites. A solution of poly(ethylene glycol) methacrylate methyl ether (*M*_n_=300, PEGMMA 300) and the ATRP catalyst was then filtered through the fiber mat to allow for SI‐ATRP grafting of hydrophilic PEGMMA 300 brush from the ATRP initiating sites on the fibers’ surface. The extent of non‐specific adsorption of a model protein on the PEGMMA 300 brush‐modified conductive fiber mat was compared with the fiber mat before the filtration‐based SI‐ATRP grafting, to evaluate the antifouling performance of the hydrophilic brush‐grafted conductive fiber mat.

## 2 Results and Discussions

### 2.1 Fabrication of the Conductive Fiber Mat

Sulfonated polystyrene‐poly(ethylene‐*ran*‐butylene)‐polystyrene (sSEBS) was co‐electrospun with a small amount of polyethylene oxide into elastomeric fiber mats using a procedure previously reported by us,^[^ ^6^ ^]^ producing randomly oriented sSEBS fibers mats.

**Figure 1:**
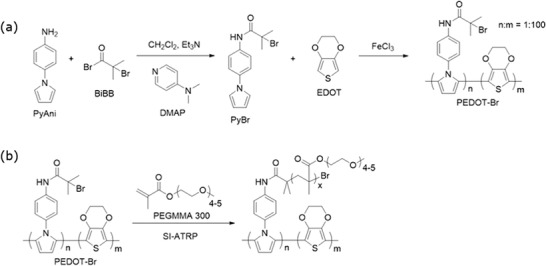
a) Synthetic scheme of ATRP initiator PyBr and copolymerization of PEDOT‐Br on the sSEBS electrospun fiber. b) Synthetic scheme of SI‐ATRP of PEGMMA 300 on the PEDOT‐Br coating.

A conducting polymer monomer, containing an ATRP initiator, N‐(4‐(1H‐pyrrol‐1‐yl)phenyl) α‐bromoisobutyramide (PyBr), was synthesized through nucleophilic acyl substitution of α‐bromoisobutyryl bromide (BiBB) with 4‐(1H‐pyrrol‐1‐yl)aniline (PyAni) (**Figure** **1**a). The sSEBS fibers were then coated with a copolymer composed of 3,4‐ethylenedioxythiophene (EDOT) and PyBr (Figure 1b) to form a conductive coating on the sSEBS (termed sSEBS/PEDOT‐Br).

**Figure 2:**
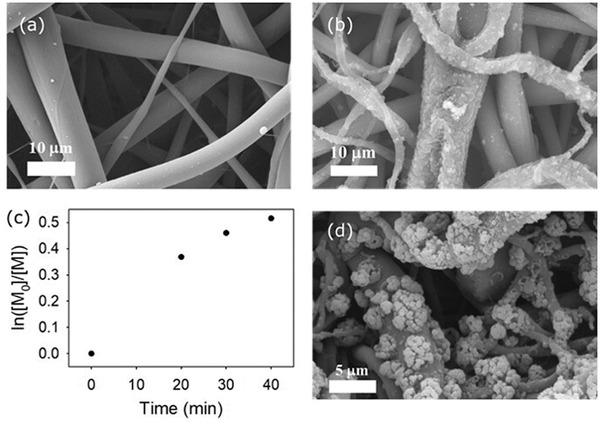
SEM image of a) sSEBS electrospun fiber and b) sSEBS/PEDOT‐Br fiber. c) Kinetic plot of SI‐ATRP polymerization of PEGMMA 300 on the sSEBS/PEDOT‐Br fiber mat initiated with PyBr as a sacrificial initiator. The fiber mat was soaked in the polymerization solution with stirring. d) SEM images of the sSEBS/PEDOT‐Br fiber after SI‐ATRP grafting with PEGMMA 300, showing the broccoli‐like, non‐uniform morphology of the grafted fiber surface.

The scanning electron microscopy (SEM) images of the sSEBS fiber mats showed a smooth morphology (**Figure** **2**a), while after the coating with P(EDOT‐*co*‐PyBr), a much rougher fiber morphology was observed (Figure 2b).

### 2.2 Grafting of PEGMMA 300

The ATRP kinetics of grafting PEGMMA 300 from PyBr initiator was first investigated in isopropyl alcohol. After optimization of the polymerization parameters (procedure given in the SI), the polymerization kinetics was found to be first order (Figure S1, Supporting Information), indicating a good control of PEGMMA 300 ATRP grafting.

Following that, the SI‐ATRP of PEGMMA 300 from sSEBS/PEDOT‐Br fiber mat was attempted with the fiber mats soaked in the polymerization solution containing PEGMMA 300, PyBr, and the ATRP catalyst. The PyBr in the solution also served as the sacrificial initiator.^[^ ^21^ ^]^ While the monomer conversion in the polymerization solution still followed the first‐order kinetics (Figure 2c), broccoli‐like structures of 1‐5 µm size were formed on the fibers’ surface (Figure 2d). In a recent report, the ATRP grafting of a related monomer, oligo(ethylene glycol) methyl ether methacrylate, resulted in a similar mushroom‐like morphology on the sSEBS fiber surface.^[^ ^19^ ^]^ The growing polymer chain initiated by the sacrificial initiator in the solution phase could also graft on an active growing site on the fiber surface through bimolecular termination. The uncontrolled covalent grafting of such high molecular weight polymer chains through bimolecular termination could form clusters of polymers, which gave the broccoli‐like structure observed on the fiber surface. Here, the SI‐ATRP was also attempted without the use of a sacrificial initiator, but no monomer conversion was observed from the aliquots of the polymerization solution. Since the initiator (PyBr) has a low molar ratio in the EDOT:EDOT‐Br comonomer feed (1:100), it is likely that the brush ATRP could not be initiated with that small fraction of the initiator sites on the fiber alone. The use of a sacrificial initiator was therefore necessary in this case.

To improve PEGMMA 300 brush grafting from the fiber surface, we developed an alternative grafting methodology. It was considered that filtering the polymerization solution through the fiber mat for the duration of the SI‐ATRP polymerization would provide a better wetting of the fibers with the polymerization solution and ensure a more uniform grafting of the brush on the fibers’ surface.

**Figure 3:**
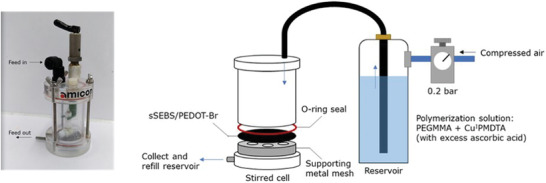
Stirred cell setup used for the SI‐ATRP filtration methodology: a photograph (left) and a schematic (right).

The filtration of a controlled volume of the polymerization solution across the membrane (under constant pressure) was examined for the grafting of PEGMMA brush from the membrane surface via SI‐ATRP. The filtration was carried out with a dead‐end filtration system (Amicon stirred cell 50 mL) (**Figure** **3**). The polymerization solution contained PEGMMA 300 monomer, copper (II) chloride, *N,N,N′,N′′,N′′*‐pentamethyldiethylenetriamine (PMDTA), and ascorbic acid, in a molar ratio of 100:0.1:1:10. The excess ascorbic acid was used here to reduce copper (II) to the copper (I) and protect the ATRP reaction from oxygen in the air.^[^ ^22^ ^]^ A sacrificial initiator (PyBr) was not added to the polymerization solution as PyBr is completely insoluble in water. 100 mL of the polymerization solution was filtered repeatedly from the reservoir through the sSEBS/PEDOT‐Br fiber mat membrane at 0.2 bar pressure. The filtered polymerization solution was collected and refilled to the reservoir to continue the grafting of PEGMMA brush. sSEBS/PEDOT‐Br fiber mats were filtered with a total of either 0.5 or 2.0 L of the PEGMMA brush polymerization solution, followed by washing the mats by filtering an excess of deionized water to remove excess reagents.

### 2.3 Chemical and Morphological Characterizations of the Filtration‐based SI_ATRP Grafted Fiber Mats

**Figure 4:**
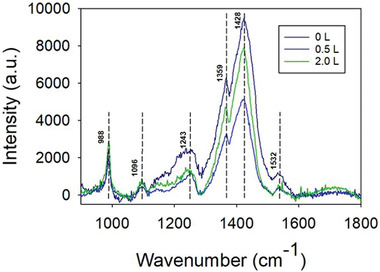
Raman spectra of sSEBS/PEDOT‐Br fiber mat after filtering with different volumes (0, 0.5, and 2.0 L) of the polymerization solution. (The Raman spectra were not normalized).

Raman spectra of the filtration‐based SI‐ATRP grafted fiber mats and the sSEBS/PEDOT‐Br fiber mat are shown in **Figure** **4**. Since the molar ratio of PyBr:PEDOT was 1:100, the PEDOT spectral features predominate in the Raman spectra of the sSEBS/PEDOT‐Br mat. Distinct PEDOT Raman peaks can be observed,^[^ ^23^, ^24^ ^]^ including asymmetric and symmetric C=C stretching (1532 and 1428 cm^‐1^), C—C stretching (1359 and 1243 cm^‐1^) in thiophene rings, C—O—C stretching (1096 cm^‐1^) and dioxyethylene ring stretching (988 cm^‐1^). These features were also found in Raman spectrum of PEDOT‐coated sSEBS in our previous work.^[^ ^6^ ^]^ After filtration of 0.5 and 2.0 L of the polymerization solution through the sSEBS/PEDOT‐Br fiber mats, the Raman spectra did not show any new distinctive peaks. However, the peak intensities of the thiophene ring stretching modes decreased while those of C—O—C modes remained at similar levels, indicating a change in the surface composition.

The peaks in Raman spectra of sSEBS/PEDOT‐Br, and grafted sSEBS/PEDOT‐Br (by filtering of either 0.5 or 2.0 L polymerization solution) were then fitted using Labspecs 6 software, with the peaks’ areas given in Table S1 (Supporting Information). The ratio of the areas of the C—O—C stretching (1096 cm^‐1^) peak to the symmetric C=C stretching (1428 cm^‐1^) increased by 0.7% and 5.2% after filtering with 0.5 and 2.0 L of the polymerization solution, respectively. The stretching peak of ethylene glycol units from the PEGMMA likely overlaps with the C—O—C stretching peak from PEDOT. A literature DFT calculation showed a Raman peak at 1106 cm^‐1^ for polyethylene glycol with four ethylene glycol units,^[^ ^25^ ^]^ supporting the notation here on the overlapping peaks between C—O—C units on PEGMMA and PEDOT on the grafted fiber surface.

**Figure 5:**
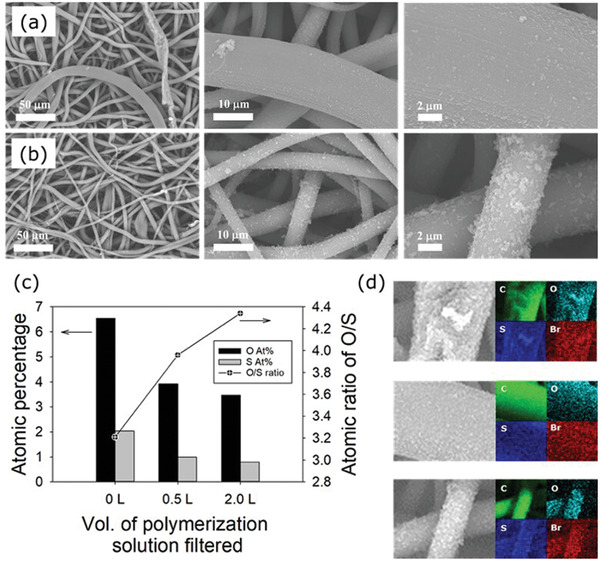
SEM images of the PEDOT/sSEBS‐Br upon filtering with a) 0.5 L and b) 2.0 L of the polymerization solution under different magnifications. c) Atomic percentage of oxygen and sulfur on the PEDOT/sSEBS‐Br after filtering with different volumes of the polymerization solution. d) Elemental mapping (carbon, oxygen, sulfur, and bromine) on the sSEBS/PEDOT‐Br fiber surface (top) and after filtering 0.5 (middle) and 2.0 L (bottom) of the polymerization solution.

The fiber mats were further examined with SEM with energy dispersive X‐ray (EDX) spectroscopy to confirm the grafting of PEGMMA brush from the sSEBS/PEDOT‐Br fiber surfaces. This time the grafted fibers had a smooth morphology with small number of rougher blobs on the surface (**Figure** **5**a,b), covering some of the rough features observed on the sSEBS/PEDOT‐Br fiber surface (Figure 1b).

The chemical changes on the sSEBS/PEDOT‐Br fiber surface upon grafting were investigated with elemental analysis through EDX spectroscopy with the details of the analysis listed in Table S2 (Supporting Information). The atomic percentage of carbon increased from 90.92% to 94.74% and 95.25% when the volume of the filtered polymerization solution increased from 0 L to 0.5 L and 2.0 L, respectively. Since PEGMMA 300 (C_12‐14_H_24‐28_O_6‐7_) has a higher carbon content than EDOT (C_6_H_6_O_2_S), the increase in the carbon content suggests that the grafting of PEGMMA brush by filtration of the polymerization solution was successful. The atomic ratio between oxygen and sulfur (O/S ratio) also increased from 3.21 to 3.96 and 4.34 with the increase in the filtration volume from 0.5 L to 2.0 L, respectively, as expected, since PEGMMA does not contain sulfur (Figure 5c). Since EDOT and PEGMMA repeating units contain 2 and 6–7 oxygen atoms, respectively, the molar ratio between the PEGMMA and PEDOT on the fiber surface could be estimated by dividing the percentage increase in the O/S ratio by 3–3.5. The molar ratio of the grafted PEGMMA brush to the PEDOT coating was therefore 0.067–0.078 and 0.101–0.117 after filtering 0.5 and 2.0 L of the polymerization solution, respectively. The elemental analysis hence indicated the continuous growth of the PEGMMA brush on the fiber surface during the filtration of the increased volume of the polymerization solution.

In addition, the atomic percentage of bromine atom on the sSEBS/PEDOT‐Br fiber throughout the brush grafting was found to be persistent (Table S2, Supporting Information), suggesting the preservation of the initiating sites on the fibers. The Br:S ratio was estimated as 0.03:1 on the sSEBS/PEDOT‐Br fiber mat surface before the filtration of the polymerization solution. Since PyBr and EDOT contain one bromine atom and one sulfur atom, respectively, the Br:S ratio could serve as an estimation of the ATRP initiating site density on the sSEBS/PEDOT‐Br fiber mat surface. The EDX elemental mapping (Figure 5d) of the sSEBS/PEDOT‐Br and grafted fiber mats also confirm the presence of bromine on the fiber surface. Moreover, the distribution of Br is uniform, which is important for the uniform grafting of the brush on the fibers, as observed in the SEM images (Figure 5a,b). Therefore, Raman spectroscopy, elemental analysis, and elemental surface mapping all confirmed the successful SI‐ATRP grafting of PEGMMA brush using the developed filtration‐based polymerization methodology.

The electrochemical properties of the grafted sSEBS/PEDOT‐Br fiber mats were examined by measuring resistance of the fiber mats and cyclic voltammetry (CV). The voltammograms, obtained in PBS buffer, (Figure S2, Supporting Information) indicate high resistance of the fibers with the redox features of PEDOT copolymer observable in the region of 0.2–0.6 V. After doping with lithium perchlorate, the sheet resistance of the fiber mats was 8.1 ± 3.6 (*n* = 5), 2.0 ± 0.4 (*n* = 5) and 17.5 ± 2.8 kΩ (*n* = 5) for sSEBS/PEDOT‐Br fiber mat filtered with 0, 0.5, and 2.0 L, respectively, which agreed with the observations from the voltammograms. When we attempted to increase the molar ratio of PyBr:EDOT in the PEDOT‐Br coating to 1:25, the sheet resistance increased to 22.4 kΩ (*n* = 10). Although a higher ATRP initiator density should lead to a more ordered brush structure through tighter packing between the individual polymer chains, and, possibly, to better antifouling performance, the higher electrical resistance was deemed undesirable for electrochemical applications. Filtration‐based ATRP grafting on sSEBS/PEDOT‐Br fibers with higher ATRP initiator density was therefore not carried out.

### 2.4 Antifouling Properties of the Grafted sSEBS/PEDOT Fiber Mats

The protein adsorption measurements were based on a method from a recent literature report.^[^ ^20^ ^]^ The antifouling performance of the sSEBS/PEDOT‐Br fiber mat filtered with 2.0 L of polymerization solution was tested with a 20 wt.% bovine serum albumin (BSA) solution. The fiber mat samples (0.25 cm^2^) were soaked in PBS buffer overnight for the PEGMMA brushes swelling to equilibrate. After that, the proteins adsorbed on the surfaces of the mats were then extracted from aqueous sodium dodecyl sulfate (SDS) solution. The concentration of the BSA protein in the SDS solution was determined by a bicinchoninic acid assay (Pierce BSA Protein Assay Kit), as described in the Experimental Section.

The absorbance of the protein assay kit was calibrated with six BSA standard solutions. The calibration curve of the protein assay showed a linear range from 0.025 mg mL^−1^ to 2.0 mg mL^−1^ (*R* ^2^ = 0.9991). The amount of BSA adsorbed on the sSEBS/PEDOT‐Br samples before filtering and after filtering with 2.0 L of polymerization solutions was determined as 2.6 ± 0.1 mg cm^‐2^ (*n* = 3) and 2.0 ± 0.2 mg cm^‐2^ (*n* = 3), respectively (with p‐value between the two data sets of 0.021, indicating a statistically significant difference between the two). The PEGMMA brush grafted through the filtration‐based grafting methodology provided a 20% reduction in the non‐specific adsorption of BSA on the fiber surface.

The short‐term chemical and thermal stabilities of the grafted sSEBS/PEDOT‐Br fiber mat samples were investigated under common physiological conditions (i.e., in PBS buffer at 20 and 40 °C for 16 h), juice filtration conditions (i.e., potassium bitartrate buffer at 20 and 40 °C for 16 h) and clean‐in‐place condition (i.e., 0.1 m sodium hydroxide at 50 °C for 1 h). The thermal and chemical stabilities were evaluated by inspecting morphological damage of the fiber mats using SEM imaging. The SEM images of the fiber mats (Table S3, Supporting Information) showed negligible morphological changes under the above conditions. It should be noted that the stability under more aggressive chemical or thermal conditions was not investigated, nor was the long‐term stability of the system. The assessment of long‐term chemical and physical stability under application‐specific conditions is a relevant topic for further study.

The above results suggest the feasibility of the developed methodology of the surface‐initiated ATRP brush grafting from porous mat substrates. This filtration grafting method has several advantages. The uniformity of the grafted brushes was much improved compared with the conventional method, and the filtration grafting method has the potential for scalability (e.g., modification of larger fiber mats or filtration membranes with a larger and/or continuous filtration module setup), allows reuse of the polymerization solution, as well as use of a water‐based solvent system.

## 3 Conclusion

A new methodology was developed for SI‐ATRP polymer brush grafting from electrospun fiber mats coated with conducting polymers by means of filtration of the polymerization solution through the fiber mat membrane. This was exemplified here by grafting PEGMMA brush from PEDOT copolymer coated onto sSEBS fiber mats. The PEGMMA polymer brush length increased with the volume of the polymerization solution filtered through the fiber mat, as confirmed by elemental analysis, SEM images, and Raman spectroscopy. Furthermore, the conductive fiber mat grafted with the hydrophilic brush reduced the non‐specific adsorption of BSA by 20%, compared to the fiber mat before filtration‐based grafting.

This new, filtration‐based SI‐ATRP methodology provides a general route to polymer brush grafting from surfaces of porous membranes.

## 4 Experimental Section

### Materials

4‐(1H‐pyrrol‐1‐yl)aniline (PyAni) and 3,4‐ethylenedioxythiohene (EDOT) were purchased from AK Scientific. 4‐Dimethylaminopyridine (DMAP), *α*‐bromoisobutyryl bromide (BiBB), polystyrene‐poly(ethylene‐*ran*‐butylene)‐polystyrene (SEBS), acetic anhydride, polyethylene oxide (*M*_n_ 100 000 g mol^−1^), poly(ethylene glycol) methacrylate methyl ether (M_n_ 300) (PEGMMA 300), copper (II) chloride, *N,N,N′,N′′,N′′*‐pentamethyldiethylenetriamine (PMDTA), ascorbic acid, phosphate‐buffered saline (PBS) tablets, lithium perchlorate, sodium hydroxide, potassium bitartrate and sodium dodecyl sulfate (SDS) were purchased from Sigma Aldrich. Dichloromethane, triethylamine, hexane, ethyl acetate, sodium bicarbonate, sodium chloride, anhydrous magnesium sulfate, chloroform, ethanol, methanol, concentrated sulfuric acid, ferric chloride, hydrogen peroxide solution (35 wt.%), isopropyl alcohol and potassium chloride were purchased from ECP Chemicals. Bovine serum albumin (BSA) was purchased from pH Scientific Limited. Pierce BCA Protein Assay Kit was purchased from Thermo Fisher Scientific.

### Synthesis of N‐(4‐1H‐pyrrol‐1‐yl)phenyl) *α*‐bromoisobutyramide (PyBr)

4‐(1H‐pyrrol‐1‐yl)aniline (PyAni) (1.00 g, 6.32 mmol) and 4‐dimethylaminopyridine (DMAP) (0.2 g, 1.64 mmol) were dissolved in dichloromethane (50 mL) in a round bottle flask (250 mL). The reaction flask was sealed with a rubber septum. Triethylamine (1.3 mL, 9.33 mmol) was added to the reaction flask with a syringe. The reaction mixture was cooled down to 0 °C with nitrogen purging for 15 min. After purging, *α*‐bromoisobutyryl bromide (BiBB) (1.17 mL, 9.47 mmol) was added into the solution dropwise. The reaction mixture was stirred in an ice bath for 30 min. The ice bath was then removed, and the reaction mixture was further stirred overnight. The reaction progress was monitored with thin‐layer chromatography (silica, aluminum backing). The product showed a UV active spot at *R*_f_ 0.56 using an eluent of hexane and ethyl acetate with a volume ratio of 8:2. After reaction completion, the reaction mixture was washed with saturated sodium bicarbonate solution (8.5 wt.%), deionized water and brine solution (20 wt.% sodium chloride). The organic layer was dried with anhydrous magnesium sulfate and filtered, followed by evaporation of residual solvent under reduced pressure. The powder obtained was washed with cold ethanol and dried in a desiccator overnight under a vacuum. The product (PyBr) obtained was a light yellow powder. Yield: 1.43 g (74%). ^1^H NMR (400 MHz, CDCl_3_, *δ*): 2.06 (s, 6H), 6.35 (dd, 2H), 7.06 (dd, 2H), 7.37 (d, 2H, *J* = 8.8 Hz), 7.60 (d, 2H, *J* = 8.8 Hz), 8.49 (s, 1H); ^13^C NMR (400 MHz, CDCl_3_, *δ*): 32.6, 63.1, 110.5, 119.3, 121.0, 121.2, 135.0, 137.6, 170.1.

### Electrospinning of Sulfonated SEBS (sSEBS) Fiber Mat

The electrospinning procedure of sSEBS fiber mat followed the previous work.^[^ ^6^ ^]^ SEBS (20 g) was dissolved in chloroform (200 mL) at 40 °C. Acetic anhydride (5 mL) was added into SEBS solution and stirred until homogenous. Concentrated sulfuric acid (1.6 mL) was then added dropwise. After stirring the reaction mixture at 40 °C for 5 h, the reaction was quenched with triethylamine (5 mL). Solvent was removed in a rotary evaporator to obtain a sticky yellow solid. The solid was gently rinsed with ethanol to remove excess reagents and further washed with ethanol in a Soxhlet extractor overnight. Washed product was dried at 40 °C in a vacuum oven overnight to remove trace amounts of ethanol to obtain sulfonated SEBS (sSEBS) as a yellow solid. Yield: 18.88 g.

sSESB (30 mL of 10 wt.%) and 1.25 wt.% polyethylene oxide (*M*_n_ 100 000 g mol^−1^) solution in chloroform was prepared for electrospinning. The electrospinning solution was loaded into a 5‐mL glass syringe with an 18G stainless needle and then placed on a syringe pump. A 20‐cm square stainless plate covered with baking paper was grounded and served as the collector for the electrospun fibers. The working distance between the needle tip and the collector was 15 cm. The needle tip was connected to a high‐voltage supply of 18 kV. The pump rate was set at 5 mL hr^−1^. The electrospinning process was carried out at 25 °C with a relative humidity of <15%. The electrospun fiber mats (sSEBS membranes) were dried at 40 °C under vacuum overnight to remove trace amounts of solvent.

### PEDOT‐Br Polymerization on sSBES Fiber Mat

The sSEBS fiber mats were soaked in a solution of EDOT (0.468 m) and PyBr (4.68 mM) in methanol for 15 min. Ferric chloride solution (1.5 m), heated to 60 °C, was added on top of the soaked membrane that was then heated at 60 °C for 1 h to complete the copolymerization of EDOT and PyBr. That afforded “PEDOT‐Br” coated on the sSEBS fiber mats (sSEBS/PEDOT‐Br). The membrane was rinsed with methanol to remove excess reagents, and then further sonicated to remove the loosely bounded coating on the fiber surface. The sSEBS/PEDOT‐Br membrane was soaked in methanol overnight and then dried at 40 °C in a vacuum oven for an additional night.

### SI‐ATRP Grafting of PEGMMA on sSEBS/PEDOT‐Br

The SI‐ATRP grafting of PEGMMA was carried out on the sSEBS/PEDOT‐Br fiber mat using the same polymerization procedure as in the kinetic study described above. The sSEBS/PEDOT‐Br fiber mat was added to the polymerization solution and the solution was degassed. Polymerization solution aliquots were withdrawn at different time points to monitor the monomer conversion. The sSEBS/PEDOT‐Br fiber mat was removed from the solution after 40 min. The fiber mat was rinsed with methanol and then soaked in methanol overnight to remove excess reagents. The fiber mat was then dried under reduced pressure and the morphology was examined with scanning electron microscopy.

### SI‐ATRP by Filtration

A polymerization solution was prepared by dissolving PEGMMA 300 (6 g) and ATRP catalyst pre‐cursor solution (2 mL, 0.01 m copper (II) chloride, and 0.1 m PMDTA in isopropyl alcohol) into deionized water (100 mL). Ascorbic acid (350 mg) was added to the polymerization solution to generate the copper (I) ATRP catalyst. The polymerization solution was immediately used after the addition of ascorbic acid.

After filling into the reservoir, the polymerization solution (100 mL) was filtered through the sSEBS/PEDOT‐Br membrane at 0.2 bar pressure with compressed air (Figure 3). The polymerization solution filtered through the membrane was collected and re‐filled into the reservoir for further filtration. The filtration continued until the total volume of polymerization solution filtered through the sSEBS/PEDOT‐Br membrane reached either 0.5 or 2 L. The excess reagents were removed from the membrane by filtering 2.0 L of deionized water through the membrane at 0.2 bar. The modified membrane sSEBS/PEDOT‐*g*‐PEGMMA was then soaked in methanol overnight and dried in a vacuum at room temperature.

### Raman Spectroscopy

Raman spectroscopy measurements were carried out using a LabRAM HR Evolution confocal Raman Microscope (Horiba, Japan) with a 785 nm laser source (100 mW) passed through a 99% neutral density filter and focused through a 50× long working distance objective lens. The scattered light was collected back through the microscope objective for 120 s and three accumulations on sSEBS/PEDOT‐Br fiber mat samples filtered with 0, 0.5, and 2.0 L of polymerization solution. The collected scattered light passed through a wide field confocal hole to a 600 l mm^−1^ diffraction grating and an air‐cooled CCD detector (−60 °C) providing a spectral resolution of ≈2 cm^‐1^. LabSpec 6 software was used for both data collection and progress. The data were baseline corrected using a multipoint third‐order polynomial background followed by peak position peak fitting using LabSpec6.

### Scanning Electron Microscopy (SEM) and Energy Dispersive X‐ray Spectroscopy (EDX)

The scanning electron microscopy was carried out with Philips FEI XL30S FEG scanning electron microscope equipped with a SiLi EDX detector. All samples were coated with 10 nm of platinum through sputtering prior to measurement. The EDX spectra were collected at 400× magnification to obtain a survey scan of the elemental analysis of the sample surface. A small area on the fiber surface was selected for the EDX mapping measurement at 15 000× magnification.

### Resistance Measurement and Cyclic Voltammetry (CV)

The cyclic voltammetry was carried out using a CH Instrument 660E. The sSEBS/PEDOT‐Br fiber mats, filtered with 0, 0.5, and 2.0 L of polymerization solution, were cut into 5 mm strips. The sample strips were connected to a potentiostat as working electrodes using an alligator clip and immersed into the electrolyte for 5 mm before the start of the measurements. A platinum mesh (6.34 cm^2^) was used as the counter electrode and an Ag/AgCl electrode in saturated KCl solution (Ag/AgCl, sat), +0.197 V versus SHE) was the reference electrode. A PBS buffer (0.01 m phosphate buffer, 0.0027 m KCl, and 0.137 m NaCl) was used as the electrolyte. The working electrodes were linearly swept from −0.6 to +0.8 V for three cycles at scan rates of 5, 25, 50, and 100 mV s^−1^. The potential sweep started from 0 V and scanned toward the negative potential in the initial scan. The final scan stopped at +0.8 V.

The sSEBS/PEDOT‐Br fiber mat samples were doped in 0.1 m lithium perchlorate through CV sweeping. The working electrodes (sSEBS/PEDOT‐Br samples) were linearly swept from −0.4 to +1.0 V for ten cycles at a scan rate of 5 mV s^‐1^. After doping, the fiber mat samples were rinsed with deionized water and dried in a desiccator overnight under a vacuum. The electrical resistance of the doped sSEBS/PEDOT‐Br fiber mats (before and after filtration with the polymerization solution) were measured with a four‐point probe conductivity meter (Jandel Engineering). The fiber mat samples were washed with an excess of deionized water and dried in a desiccator overnight before the resistance measurement. The sheet resistance *R*_s_ of the fiber mat samples was calculated through the following formula:

(1) $$\begin{array}{c} R_{s}=\frac{\pi }{\ln2}\frac{U}{I} \end{array}$$

where *U* is the measured potential (*V*) and *I* is the input current.

### Protein Adsorption Test

The protein adsorption test was based on a literature report.^[^ ^20^ ^]^ The sSEBS/PEDOT‐Br fiber mats, filtered with 0 and 2.0 L of polymerization solution, were cut into 0.5 cm × 0.5 cm samples (*n* = 3). All samples were soaked in PBS buffer overnight before the tests. A 20 wt.% BSA solution in PBS buffer was prepared as the fouling solution. The fiber samples were soaked in the fouling solution (1 mL) at room temperature for 24 h. After removing from the fouling solution, the fiber mat samples were dipped into fresh PBS buffer three times to remove the loosely bound proteins from the sample surface. The fiber mat samples were then transferred into a micro‐centrifuge tube (1.5 mL) with 1 wt.% SDS solution in PBS buffer (1 mL). The micro‐centrifuge tubes were sonicated for 20 min to extract the BSA protein adsorbed on the fiber surface.

The protein concentration in the SDS extract solution was determined by Pierce BCA assay kit. A series of BSA solutions (2, 1, 0.5, 0.25, 0.125, 0.025 mg mL^−1^) and a blank sample were prepared in PBS buffer containing 1 wt.% SDS. The working solution of the assay kit was prepared according to the manufacturer's instructions. The working solution (2 mL) was then transferred to a 15 mL centrifuge tube. Each of calibration standards and the SDS extracts from fouled samples (0.1 mL) were added into the working solution and mixed well with gentle shaking. The working solutions were then incubated at 37 °C for 30 min. The absorbance at 526 nm was measured with a UV–vis spectrometer. The absorbance of calibration standards and SDS extracts of fouled samples was subtracted from the absorbance of the blank sample. Protein concentrations determined by this method were used to calculate protein per unit area in the membrane samples (expressed as mg cm^‐2^). The statistical significance of the results obtained from the tests on the sSEBS/PEDOT‐Br fiber mats filtered with 0 and 2.0 L of polymerization solution was evaluated with an unpaired t‐test with two tails at a significant level of 0.05.

## Conflict of Interest

The authors declare no conflict of interest.

## Supporting information

Supporting Information

## Data Availability

The data that support the findings of this study are available from the corresponding author upon reasonable request.
