# Supplementary material for: Grafting of Porous Conductive Fiber Mats with an Antifouling Polymer Brush by Means of Filtration‐Based Surface Initiated ATRP
Source: Macromol Rapid Commun. 2023 Apr 6;46(8):2300069. doi: 10.1002/marc.202300069 (PMC12004905; doi:10.1002/marc.202300069)
Supplement: Supplementary file 1 — Supporting Information [file MARC-46-2300069-s001.pdf]

Macromolecular Rapid Communications

Supporting Information

for *Macromol. Rapid Commun.*, DOI 10.1002/marc.202300069

Grafting of Porous Conductive Fiber Mats with an Antifouling Polymer Brush by Means of Filtration-Based Surface Initiated ATRP

Sheung-Yin Li, Benjamin S. Schon and Jadranka Travas-Sejdic**

Supporting Information

Grafting of Porous Conductive Fiber Mats with an Antifouling Polymer Brush by Means of Filtration-based Surface Initiated ATRP

Sheung-Yin Li, Benjamin S. Schon and Jadranka Travas-Sejdic**

Kinetic studies of ATRP with PyBr: A solution of PEGMMA 300 (3 g, 10 mmol), PyBr, copper (II) chloride and PMDTA, in the molar ratio of 400:4:0.1:1, was prepared in isopropyl alcohol, and mixed with the solution of copper (II) chloride (6 mg/mL, 44.55 mM), PMDTA (2 v/v%, 0.0958 mM) and PyBr (15 mg/mL, 49.07 mM) in isopropyl alcohol, making a total of 4.75 mL solution. The glass vial was then sealed with a rubber septum and put in a water bath at room temperature (20 °C). The polymerization solution was then degassed with nitrogen for 15 minutes.

The ascorbic acid solution (10 mL, 0.1 M) was freshly prepared in DMF to which hydrogen peroxide solution (45 µL, 35 w/v%) was added. The ascorbic acid solution (0.25 mL) was injected into the glass vial with the polymerization solution to start the polymerization. At a number of time point, polymerization solution aliquots (0.1 mL) were withdrawn and diluted with deuterated methanol. The monomer concentration in the polymerization solution at different time point was measured by nuclear magnetic resonance spectroscopy using DMF (δ = 8.0 ppm) as the internal standard.

Figure S1. Kinetic plot of the ATRP of PEGMMA 300 monomer initiated with PyBr in isopropyl alcohol. The linear range was valid from 0 to 40 minutes, which indicated the ATRP polymerization is well controlled within this time period.

Table S1. Peak areas of corresponding stretching modes in the Raman spectra of sSEBS/PEDOT-Br fiber mats filtered with different volumes of the polymerization solution.

Stretching mode	Wavenumber (cm-1)	Polymerization solution filtered		
		0 L	0.5 L	2.0 L
C=C asymmetric	1532	863.1	286.6	400.4
C=C symmetric	1428	8204.8	4951.4	7372.5
C-C asymmetric	1359	4908.9	2308.3	3643.6

C-C symmetric	1243	1746.1	748.7	1181.9
C-O-C stretching	1096	669.9	407.0	633.0
Dioxyethylene ring	988	2429.3	1868.6	2704.9

Table S2. Atomic ratio of different elements detected in the EDX spectra of the sSEBS/PEDOT-Br fiber mats filtered with 0 L, 0.5 L and 2.0 L of the polymerization solution.

Element	Atomic ratio (%)		
	0 L	0.5 L	2.0 L
Carbon	90.92	94.74	95.25
Oxygen	6.54	3.92	3.47
Sulfur	2.04	0.99	0.80
Bromine	0.06	0.06	0.14
Iron	0.20	0.20	0.21
Chlorine	0.25	0.08	0.13

Figure S2. Cyclic voltammograms of sSEBS/PEDOT-Br fiber mats filtered with 0 L, 0.5 L and 2.0 L of the polymerization solution in PBS buffer at a scan rate of 5 mV s^{-1}.

Table S3. SEM images of sSEBS/PEDOT-Br fiber mats filtered with different volumes of polymerization solution after the thermal and chemical stability tests.

	Volume of polymerization solution filtered		
	0 L	0.5 L	2.0 L

0.1 M NaOH (pH =13), 50 °C, 1 hour	Sat. K bitartrate buffer (pH 3.55), 40 °C, 16 hours	PBS buffer (pH 7.4), 40 °C, 16 hours	Sat. K bitartrate buffer (pH 3.55), 20 °C, 16 hours	PBS buffer (pH 7.4), 20 °C, 16 hours
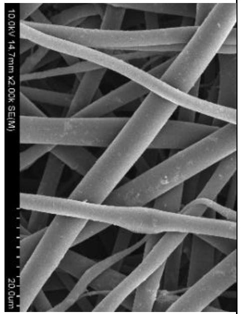	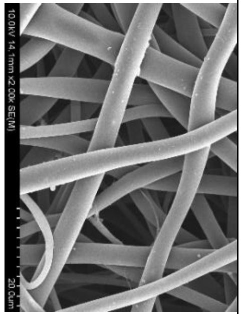	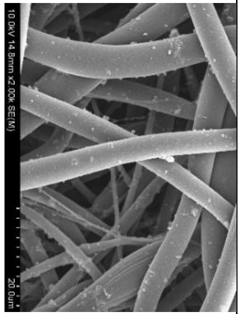	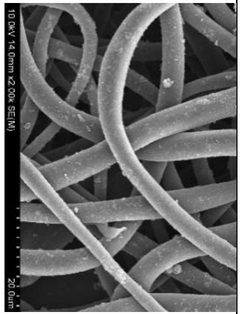	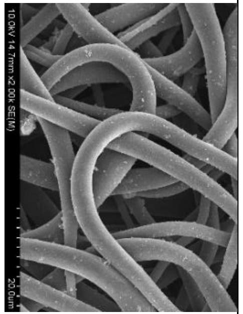
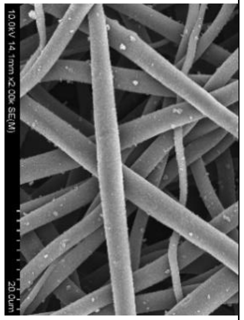	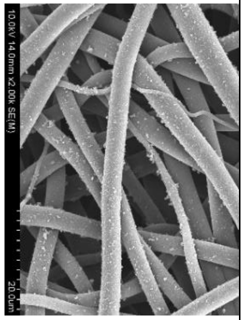	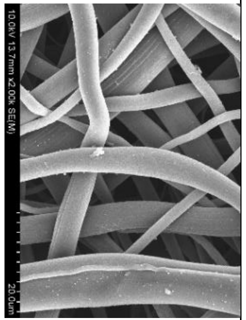	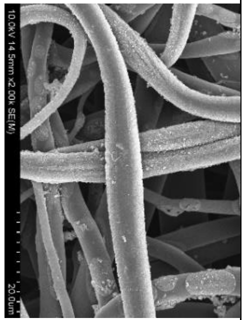	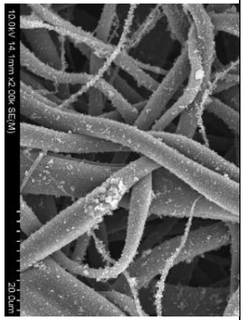
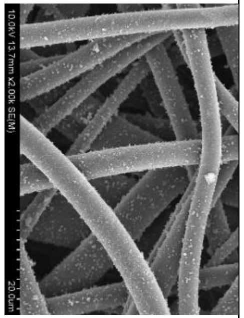	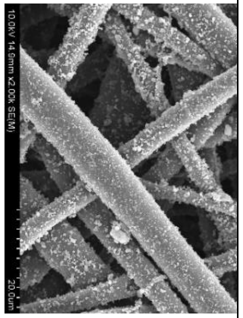	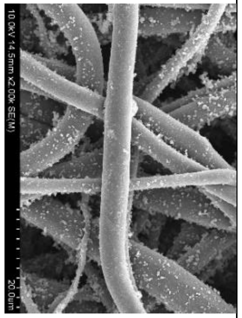	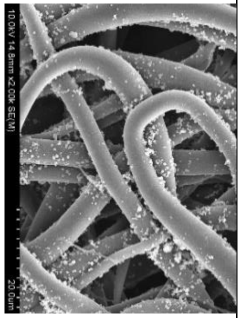	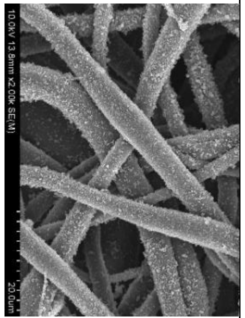
